# Investigating the Influence of Glycerol on the Utilization of Glucose in *Yarrowia lipolytica* Using RNA-Seq-Based Transcriptomics

**DOI:** 10.1534/g3.119.400469

**Published:** 2019-10-18

**Authors:** Patrice Lubuta, Mhairi Workman, Eduard J. Kerkhoven, Christopher T. Workman

**Affiliations:** * Department of Biotechnology and Biomedicine, Technical University of Denmark, 2800 Kgs. Lyngby, Denmark,; † Department of Biology and Biological Engineering, Division of Systems and Synthetic Biology, Chalmers University of Technology, SE-412 96 Gothenburg, Sweden, and; ‡ Novo Nordisk Foundation Center for Biosustainability, Chalmers University of Technology, SE-412 96 Gothenburg, Sweden

**Keywords:** Yarrowia lipolytica, quantitative physiology, carbon repression, transcriptomics, RNA-Seq

## Abstract

Glycerol is considered as a promising substrate for biotechnological applications and the non-conventional yeast *Yarrowia lipolytica* has been used extensively for the valorization of this compound. Contrary to *S. cerevisiae*, *Y. lipolytica* seems to prefer glycerol over glucose and it has been reported previously that the presence of glycerol can suppress the consumption of glucose in co-substrate fermentations. Based on these observations, we hypothesized glycerol repression-like effects in *Y. lipolytica*, which are converse to well described carbon repression mechanisms ensuring the prioritized use of glucose (*e.g.*, in *S. cerevisiae*). We therefore aimed to investigate this effect on the level of transcription. Strains varying in the degree of glucose suppression were chosen and characterized in high-resolution growth screenings, resulting in the detection of different growth phenotypes under glycerol-glucose mixed conditions. Two strains, IBT and W29, were selected and cultivated in chemostats using glucose, glycerol and glucose/glycerol as carbon sources, followed by an RNA-Seq-based transcriptome analysis. We could show that several transporters were significantly higher expressed in W29, which is potentially related to the observed physiological differences. However, most of the expression variation between the strains were regardless of the carbon source applied, and cross-comparisons revealed that the strain-specific carbon source responses underwent in the opposite direction. A deeper analysis of the substrate specific carbon source response led to the identification of several differentially expressed genes with orthologous functions related to signal transduction and transcriptional regulation. This study provides an initial investigation on potentially novel carbon source regulation mechanisms in yeasts.

Glycerol, a by-product from the biodiesel production is considered as a promising substrate for biotechnological applications. The biodiesel industry increased rapidly in the European Union and the U.S. over the last fifteen years, leading to an increased availability of crude glycerol and a drastic decrease of its market price (Valerio *et al.* 2015). The use of glycerol by microbial fermentation makes high efficient production strains (so-called *cell factories*) indispensable. *Saccharomyces cerevisiae* is among yeasts the most established cell factory applied, however, its natural capacity to utilize this substrate is limited (Klein, Swinnen, Thevelein, and Nevoigt 2017). In contrast, several other yeast species, *e.g.*, *Pachysolen tannophilus*, *Pichia pastoris*, *Cyberlindnera jadinii* or *Yarrowia lipolytica*, are naturally superior glycerol users (Klein, Islam, Knudsen, Carrillo, Swinnen, *et al.* 2016). The oleaginous yeast *Y. lipolytica* has gathered attention in recent years, especially due to its ability to produce economically interesting compounds (Liu, Ji, and Huang 2015). Growth rates of *Y. lipolytica* on glycerol exceed levels of 0.4 h^-1^ (Klein", Islam, Knudsen, Carrillo, Swinnen, *et al.* 2016) and various attempts have aimed to convert glycerol into value-added products (Rywińska, Juszczyk, Wojtatowicz, Robak, Lazar, *et al.* 2013).

Glycerol metabolism has been extensively studied in *S. cerevisiae* (Klein, Swinnen, Thevelein, and Nevoigt 2017). Both species, *S. cerevisiae* and *Y. lipolytica* are using the glycerol-3-phosphate pathway in order to metabolize glycerol (Dulermo and Nicaud 2011; Sprague and Cronan 1977; Pavlik, Simon, Schuster, and Ruis 1993), however, several differences in glycerol uptake, the presence of metabolic enzymes and carbon source regulation exist. In contrast to *S. cerevisiae*, *Y. lipolytica* appears to prefer glycerol over glucose as a source of carbon and energy: It could be shown in single carbon cultivations that growth rates on glycerol are higher than those on glucose, and additionally, that the consumption of glucose is suppressed in glucose-glycerol co-cultivations (Workman, Holt, and Thykaer 2013; Mori, Iwama, Kobayashi, Horiuchi, Fukuda, *et al.* 2013; Yuzbasheva, Mostova, Andreeva, Yuzbashev, Fedorov, *et al.* 2018). Interestingly, glucose consumption is restored after glycerol depletion. These observations point to carbon regulation mechanisms allowing *Y. lipolytica* the prioritized use of glycerol. The underlying mechanisms have not been elucidated yet, but must differ drastically from well-known carbon catabolite repression (CCR) mechanisms (*e.g.*, in *S. cerevisiae* or *E. coli*) that ensure the prioritized use of glucose (Gancedo 1998; Brückner and Titgemeyer 2002). For instance, in *S. cerevisiae* genes related to glycerol uptake (*STL1*) and catabolism (*GUT1*, *GUT2*) are repressed under glucose and derepressed after its depletion when growth occurred on non-fermentable carbon sources (Grauslund, Lopes, and Rønnow 1999; Grauslund and Rønnow 2000; Ferreira, van Voorst, Martins, Neves, Oliveira, *et al.* 2005).

This study provides an initial investigation on potentially novel carbon source regulation mechanism in the non-conventional yeast *Y. lipolytica*. Known carbon regulatory mechanisms act on the level of transcription, and therefore, our approach aimed to investigate *Y. lipolytica*’s transcriptome. While so far glycerol mediated repression of glucose utilization has only been described for the haloarchaeon *Haloferax volcanii* (Sherwood *et al.* 2009), it could be shown that n-alkane utilization in *Y. lipolytica* is transcriptionally repressed by glycerol (Iida, Sumita, Ohta, and Takagi 2000; Iida, Ohta, and Takagi 1998; Mori, Iwama, Kobayashi, Horiuchi, Fukuda, *et al.* 2013). Interestingly, the above mentioned glycerol induced suppression of glucose consumption in co-substrate cultivations seems to be strain dependent. While most strains showed glycerol repression-like effects, some strains were able to use glycerol and glucose simultaneously. We therefore tried to gain insights from the analysis of these strains: in initial experiments, the growth physiology was investigated by high-frequency biomass measurements in order to identify diauxic shift-like events during mixed substrate cultivations. The strains IBT and W29 were selected and grown in chemostats using glycerol, glucose and a glycerol-glucose blend as carbon sources. Samples were taken and analyzed by RNA-Seq based transcriptomics in order to compare the transcriptional profiles.

## Materials and Methods

### Yeast strains and media

Three wild type *Y. lipolytica* strains were used throughout this study: *Y. lipolytica* W29 (CLIB 89) and *Y. lipolytica* H222 (CLIB 80) were obtained from CIRM-Levures strain collection, Institute National de la Recherche Agronomique (INRA, France). *Y. lipolytica* IBT 446 (hereafter referred to as “IBT”) was obtained from the culture collection of the Department of Biotechnology and Biomedicine, Technical University of Denmark (DTU). For long-term storage, strains were grown in YPD liquid media (1% yeast extract, 2% glucose, 2% peptone) and kept at -80° in 17% (v/v) glycerol. YPD plates were used for short-term storage and the strains were grown for 2 days at 30°. YPD plates were stored at 4°. All cultivation experiments were performed in defined minimal media as described in (Workman, Holt, and Thykaer 2013).

### Microscale cultivations

A microscale fermentation system (BioLector, m2p-Labs GmbH) was used to screen for growth differences when varying glycerol-glucose blends were used as carbon sources. Cultivations took place in 48-well microtiter plates (MTP-48-B Flowerplates, m2p-Labs GmbH) with a working volume of 1.5 ml and 1000 rpm shaking speed. The temperature was maintained at 30°, and humidity control was active to reduce evaporation. Online monitoring of biomass accumulation was achieved by light scattering measurement at 620 nm approximately every 3 min. Table 1 shows the used glycerol and glucose concentrations. Precultures were grown in shake flasks using defined minimal media and 20 g L^-1^ glycerol as the carbon source. Cells were harvested during mid exponential phase, and washed to remove residual substrate. Experiments were conducted with at least 4 replicates and in independent plate runs.

**Table 1:** Glycerol and glucose concentrations used in microscale growth experiments

#	Glycerol: Glucose Ratio	Glycerol concentration [mole L^-1^]	Glucose concentration [mole L^-1^]
1.	1: 0	0.054	0
2.	2: 1	0.036	0.018
3.	1: 1	0.027	0.027
4.	1: 2	0.018	0.036
5.	1: 4	0.011	0.043
6.	0: 1	0	0.054

### Chemostat cultivations

Chemostat cultivations were carried out in order to generate biomass samples used for mRNA extraction. Cultivations were conducted in fully instrumented and automatically controlled 1 L BIOSTAT Q plus fermenters (Sartorius Stedim Biotech S.A) with a working volume of 0.5 L. Cells were grown in batch mode until late exponential phase (determined by CO_2_ exhaust gas measurement) before to the continuous mode was initiated. Liquid in and out flows were controlled gravimetrically. Carbon limited conditions were applied and the dilution rate was adjusted to D = 0.1 h^-1^. Three experimental conditions have been tested: glycerol 10 g L^-1^ (≈ 0.11 mole L^-1^), glucose 10 g L^-1^ (≈ 0.06 mole L^-1^) and a mix of glycerol 5 g L^-1^ (≈ 0.05 mole L^-1^) and glucose 5 g L^-1^ (≈ 0.03 mole L^-1^). All cultivations were applied in triplicates resulting in 18 total chemostat cultivations.

The biomass concentration was determined by cell dry weight estimation using 0.45 μm nitrocellulose filters (Sartorius Stedium) for broth filtration and microwave desiccation (150 W for 20 min). HPLC analysis was used to quantify substrate concentrations. The fermentation broth was filtered and compounds were separated by an Aminex HPX-87H column (Bio-Rad) prior the detection via RI detector. Off-gas analysis was carried out by mass spectrometry using a Prima PRO Process Mass Spectrometer (Thermo Fisher Scientific) quantifying the exhaust gas composition. Biomass samples were taken under steady state conditions (after 3 residence times). The broth was centrifuged in 2 ml aliquots and cell pellets immediately frozen in liquid nitrogen. Biomass samples were kept at -80° until further use.

### RNA extraction and sequencing

Cell pellets were disrupted and homogenized by bead-milling in a TissueLyser (Quiagen) and the use of metal beads. RNA extraction was carried with the RNeasy Plus Mini Kit (cat. nos. 74134, Quiagen) according the standard protocol. Samples were barcoded, multiplexed and sequenced using a HiSeq 4000 instrument (illumina) in paired end mode. Reads with a length of 150 base pairs were generated. The raw sequencing reads are available on the NCBI Sequence Read Archive (SRA) under the BioProject ***PRJNA437435*** (see Data Availability section below).

### Transcriptome data analysis

Raw reads were demultiplexed with the Barcode Splitter tool from the FASTX toolkit version 0.0.14 (Hannon 2010). The raw reads were subsequently quality controlled with the FastQC tool version 0.11.5 (Andrews 2010) and quality trimmed with Trimmomatic version 0.36 (Bolger, Lohse, and Usadel 2014). Read mapping and quantification was carried out with the Subread package (Liao, Smyth, and Shi 2013) using the W29 genome as a reference (GenBank assembly accession: GCA_001761485.1) (Magnan, Yu, Chang, Jahn, Kanomata, *et al.* 2016). In order to facilitate the comparability between W29 gene identifiers (YALI1_ID) and the older CLIB 122 (GenBank assembly accession: GCA_000002525.1) identifiers (YALI0_ID), we provide both identifiers in every table.

Raw read counts have been converted into transcripts per million (TPM) according Wagner, Kin, and Lynch (2012), to compare the expression of different genes across the samples. A differential gene expression analysis was carried out using the edgeR package (Robinson, McCarthy, and Smyth 2010) for importing, filtering and normalizing raw count data and the limma package for linear modeling (Law, Chen, Shi, and Smyth 2014).

Several linear models have been used throughout the study: In order to extract the strain effect we used a model describing the expression as function of strain effect (s) and carbon source condition effect (c): y=sx+cx+ϵ. The strain term was categorical while the condition term was assumed to be ordinal resulting in a linear coefficient and a quadratic coefficient. Further, to analyze strain-specific responses to the applied carbon sources, cross-comparisons between samples have been carried out. Therefore, the strain and condition factors were combined into one factor (*e.g.*, IBT_glycerol) and comparisons of interest were extracted as contrasts. Finally, to investigate the influence of the different carbon sources across the two strains, we formulated three models with separate factors for glucose (c_glu_) and glycerol (c_gly_) (present *vs.* not-present). In order to extract genes responding to the presence of glucose in both strains we formulated model 1: $y=\mathrm{sx}+c_{\mathrm{glu}}x+\epsilon$. To extract genes responding to the presence of glycerol in both strains we formulated model 2: $\;y=\mathrm{sx}+c_{\mathrm{gly}}x+\epsilon$. Finally, to extract genes differently responding in the two strains we formulated model 3: $=sx+c_{\mathrm{gly},\mathrm{IBT}}x+c_{\mathrm{glu},W29}x+\epsilon$, where we specifically modeled the factor glycerol and IBT, and the factor W29 and glucose.

### Gene set analysis

Gene Ontology (GO) term annotations of the *Y. lipolytica* W29 genome (biosample: SAMN04088558) were assigned by Blast2GO (Conesa, Gotz, Garcia-Gomez, Terol, Talon, *et al.* 2005) using the provided fungi reference database and InterProScan (Jones, Binns, Chang, Fraser, Li, *et al.* 2014) using default settings. GO term annotations are provided in File S2. The Piano R-package was used for gene-set analyses (GSA) (Väremo, Nielsen, and Nookaew 2013). Only gene sets with more than 5 and less than 500 genes were included. Piano’s consensus gene-set analysis function was used to condense results from several different GSA methods.

### Data availability

All *Y. lipolytica* strains are available upon request. File S1 contains detailed descriptions of all supplemental files. Raw sequence data (RNA-Seq data) are available on the NCBI Sequence Read Archive (SRA) under the BioProject ***PRJNA437435*** and accession numbers are listed in File S1. Table S1 contains a list of investigated transporters and Table S2 contains a list with glycerol metabolic genes. File S2 contains Gene Ontology (GO) annotations. File S3 contains the raw RNA-Seq count values while File S4 contains count values converted into transcripts per million (TPM). File S5 contains gene-level statistics of a model describing the expression as a function of strain and condition. File S6 contains gene-level statistics of cross comparisons between samples of a strain and File S7 contains gene-level statistics of the three models investigating the carbon source response across the two strains (see Materials & Methods: Transcriptome data analysis). Figure S1 shows the substrate consumption of *Y. lipolytica* strains IBT and W29 under glycerol-glucose mixed conditions. Figure S2 shows expression levels of differentially expressed genes as identified by model 3. Supplemental material available at figshare: https://doi.org/10.25387/g3.8335217.

## Results and Discussion

### Glycerol-glucose mixed cultivations revealed physiological differences between Y. lipolytica strains W29 and IBT

**Figure 1:**
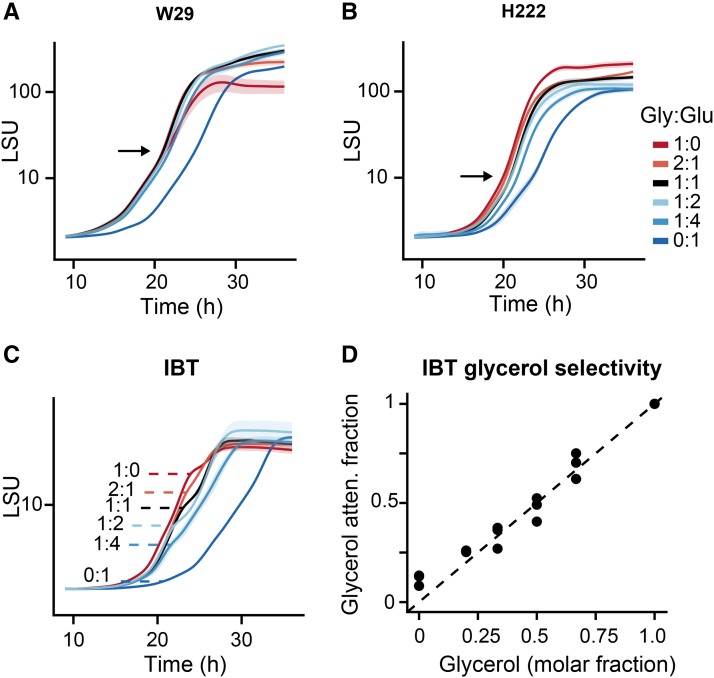
Growth profiling in microscale cultivations with different glycerol and glucose ratios. Biomass accumulation was monitored online by measurements of scattered light (LSU: Light Scattering Unit). (A-C): Growth profiles of W29, H222 and IBT. (D): Correlation between glycerol molar fraction and glycerol attenuation fraction (first growth phase) in growth experiments of strain IBT.

Previous results showed that the *Y. lipolytica* strain IBT exhibits a sequential substrate utilization of glycerol and glucose, while the strains W29 and H222 exhibit a higher degree of co-consumption (Lubuta *et al.* 2018, manuscript under review). Based on these observations, we postulated that carbon repression-like mechanisms ensure the prioritized use of glycerol and that these mechanisms are strain dependent in *Y. lipolytica*. In a first attempt to investigate these phenomena, it should be determined if these strain dependent substrate utilization phenotypes have an effect on growth when glycerol-glucose mixtures are applied. Microscale cultivations with high-frequency biomass measurements (approximately every 3 min) were used to detect small changes in the biomass accumulation (diauxic shift-like events). Since *Y. lipolytica* grows faster on glycerol (µ = 0.30 h^-1^) than on glucose (µ = 0.24 h^-1^) (Workman, Holt, and Thykaer 2013), two growth phases should be visible for strains exhibiting a sequential consumption, whereas only one growth phase should be present if strains exhibiting co-consumption. Additionally, a short second lag phase between the consumption of glycerol and glucose was observed by strains with sequential uptake. The three *Y. lipolytica* strains W29, H222 and IBT were tested on six different glycerol-glucose ratios and growth profiles are shown in Figure 1. Experiments were conducted with at least 4 (max. 5) replicates and in independent plate runs.

**Figure 2:**
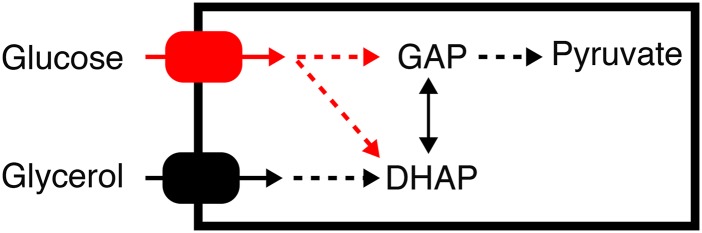
Hypothesis for the observed phenotypical differences between IBT and W29. Glucose and glycerol catabolic routes are connected over the common intermediate DHAP (see also Figure 4). We hypothesized glycerol repression-like effects in IBT preventing the simultaneous consumption of glucose in the presence of glycerol. Potential targets of this repression are glucose uptake or genes of the upper glycolysis (both in red). GAP: glyceraldehyde-3-phosphate. DHAP: dihydroxy-acetonephosphate.

All three strains showed an initial lag phase, which was longer when glucose was the solely carbon source. This was also the case when pre-cultures were grown on glucose instead of glycerol (data not shown). After the initial lag phase all strains grew exponentially. The high-resolution growth profiles revealed two types of transitions in the biomass accumulation: All growth curves of W29 and H222 showed a modest increase in the growth half-way through their cultivation time (20-25 h, indicated by black arrows in Figure 1A and B). However, this transition was observable under all conditions (including the 1:0 and 0:1 ratios), and therefore, a specific response to the varying ratios was excluded. We assumed this transition indicates morphological changes, since hyphae formation was detected by microscopy (data not shown). Interestingly, another type of transition was observable in cultivations with IBT: Here, two distinct growth phases were distinguishable, whereby the first one increased in its length the more glycerol was available (dashed line in Figure 1C). A significant correlation was observed between the proportion of biomass generated before an observable diauxic shift (the glycerol attenuation fraction) and the molar fraction of glycerol in the media (Figure 1D). In contrast, it was not possible to link the substrate molar fractions to the transition phases in W29 and H222 cultivations. These results support a sequential substrate utilization by IBT which has a direct effect on the growth. The sequential utilization pattern was also observed in additional shake flask cultivations (Figure S1). Based on these findings we formulated the hypothesis that genes related to glucose utilization are subject to a glycerol induced repression in IBT, while this effect is absent or reduced in W29 (Figure 2). To test this hypothesis, we selected the strains IBT and W29 for chemostat cultivations and a subsequent transcriptome analysis. The gene expression data were used to investigate if observed physiological differences were linked to differences in gene expression.

### Chemostat cultivations revealed lower respiration rates in IBT

Chemostat cultivations with the strains *Y. lipolytica* IBT and W29 were conducted in order to gain biomass samples for a subsequent transcriptome analysis. Three different conditions were applied: defined minimal media with either glycerol, glucose or a glycerol-glucose blend (each condition in triplicates). The chemostats were carbon limited with a dilution rate of 0.1 h^-1^. Transcriptomes provided during growth on single carbon sources were then compared with the glycerol-glucose mixed condition revealing potential differences in carbon source regulation between the two strains. Table 2 shows the main physiological parameters of the chemostat experiments. Due to carbon limited conditions, substrate concentrations in the bioreactor were not detected throughout all conditions (0 g L^-1^). Specific substrate consumption rates in mmole substrate gDW^-1^ h^-1^ were roughly twice those for glycerol compared to glucose, since glycerol has only half of the molecular weight of glucose (92.09 g mole^−1^ *vs.* 180.16 g mole^−1^) resulting in the double amount of moles used in the cultivations. Since the biomass concentration of W29 under mixed conditions was slightly lower than in the other cultivations, calculations led to slightly higher specific substrate consumption rates (q_Glu_ and q_Gly_). Specific oxygen consumption rates q_O2_ and carbon dioxide production rates q_CO2_ of both strains varied throughout the applied conditions. In glucose cultivations oxygen consumption and carbon dioxide production had nearly the same values which is reflected by a respiratory quotient (RQ) of close to one. In contrast, oxygen consumption was higher than the carbon dioxide production when grown under glycerol, giving a RQ of 0.67 (IBT) and 0.69 (W29). In mixed substrate cultivations oxygen consumption rates and carbon dioxide production rates showed values in between the single carbon cultivations, resulting in RQ values of 0.84 (IBT) and 0.85 (W29). Interestingly, q_O2_ and q_CO2_ were generally higher in the W29 cultivations throughout all conditions, which could indicate that W29 has a more active oxidative phosphorylation. However, the biomass yields Y_sx_ for IBT and W29 were similar throughout almost all conditions: roughly 65% of the carbon went into biomass (cmole cmole^-1^). For both strains carbon dioxide yields Y_sc_ were higher on glucose compared to the other conditions. Only carbon yields from W29 cultivations under glucose add up to one. In the other cultivations some carbon was unaccounted (approximately 10%), indicating the secretion of undetected by-products.

**Table 2:** Physiological parameters of the carbon limited chemostat experiments at steady state. The strains IBT and W29 have been cultivated on glucose, glycerol and a glucose-glycerol mix (each condition in triplicates) with a dilution rate of 0.1 h^-1^. RQ: respiratory quotient. DO: dissolved oxygen

	IBT glucose	IBT glycerol	IBT glucose/glycerol	W29 glucose	W29 glycerol	W29 glucose/glycerol
**Biomass conc. (g L^-1^)**	5.3 ± 0.2	5.3 ± 0.3	5.4 ± 0.4	5.1 ± 0.2	5.3 ± 0.0	4.6 ± 0.1
**q_Glu_ (mmole gDW^-1^ h^-1^)**	−1.04 ± 0.03	0.00 ± 0.00	−0.51 ± 0.04	−1.08 ± 0.04	0.00 ± 0.00	−0.61 ± 0.02
**q_Gly_ (mmole gDW^-1^ h^-1^)**	0.00 ± 0	−2.04 ± 0.11	−1.01 ± 0.07	0.00 ± 0.00	−2.05 ± 0.01	−1.19 ± 0.03
**q_O2_ (mmole gDW^-1^ h^-1^)**	−1.67 ± 0.05	−2.17 ± 0.11	−1.75 ± 0.28	−2.23 ± 0.06	−2.49 ± 0.07	−2.39 ± 0.12
**q_CO2_ (mmole gDW^-1^ h^-1^)**	1.81 ± 0.02	1.46 ± 0.06	1.46 ± 0.22	2.41 ± 0.02	1.71 ± 0.06	2.03 ± 0.07
**Y_sx_ (cmole cmole^-1^)**	0.64 ± 0.02	0.66 ± 0.04	0.66 ± 0.04	0.62 ± 0.02	0.65 ± 0.0	0.55 ± 0.02
**Y_sc_ (cmole cmole^-1^)**	0.29 ± 0.01	0.24 ± 0.02	0.24 ± 0.03	0.37 ± 0.02	0.28 ± 0.01	0.28 ± 0.01
**RQ (-)**	1.08 ± 0.04	0.67 ± 0.03	0.84 ± 0.02	1.08 ± 0.02	0.69 ± 0.01	0.85 ± 0.01
**DO (%)**	66 ± 12	45 ± 3	59 ± 2	51 ± 3	40 ± 1	55 ± 2

Carbon source concentration for all substrates at steady state: 0 g L^-1^.

### Evidence for differences in physiology of carbon utilization are observed in mRNA levels of targeted genes

RNA samples obtained from chemostat cultivations were sequenced and resulting reads have been quantified using the W29 genome (GenBank assembly accession: GCA_001761485.1) as a reference. In order to gain insight into strain similarities at the nucleotide level, the number of SNPs identified by the RNA-Seq data were quantified. Compared to the W29 reference genome, 155 SNPs were found in W29 and 11585 SNPs in IBT. To investigate if any transcripts were measured that were not in the reference genome, unmapped reads were collected and *de novo* assembled using SPAdes version 3.13.1 (Bankevich, Nurk, Antipov, Gurevich, Dvorkin, *et al.* 2012). There was no evidence for transcripts which are not present in the reference genome. Raw RNA-Seq count values are provided in File S3. The genome-wide expression data were analyzed by two approaches: in the targeted analysis genes directly involved in glycerol and glucose metabolism and transport have been investigated, while in the explorative approach linear modeling was used to systematically analyze the effects caused by the experimental factors. YALI1 gene identifiers have been used throughout this study, but YALI0 identifiers are provided in tables to facilitate comparability with the older CLIB 122 YALI0 identifiers (GenBank assembly accession: GCA_000002525.1).

#### Significant strain differences were observed between glucose and glycerol transporters:

Only a few studies have addressed glucose and glycerol uptake in *Y. lipolytica*. One attempt to decipher sugar transport mechanisms in *Y. lipolytica* resulted in the identification of 24 proteins related to sugar transport (Lazar, Neuvéglise, Rossignol, Devillers, Morin, *et al.* 2017). The authors showed that these putative sugar porters are distributed among six different clusters (class A to F) in a phylogenetic analysis including proteins from *Y. lipolytica*, *S. cerevisiae* and *Kluyveromyces lactis*. Further, it could be shown that six out of the 24 identified proteins function as hexose transporters (therefore named *Yarrowia Hexose Transporter*: Yht1 to Yht6 by the authors) and among them Yht1 and Yht4 seem to be most important for glucose uptake. In the present study, we used the nomenclature presented by Lazar *et al.* (2017) and investigated the expression levels of all identified transporters (Table S1). Furthermore, there is strong evidence that glycerol uptake in *Y. lipolytica* is mediated by an ortholog to *S. cerevisiae* aquaglyceroporin Fps1 (Klein, Islam, Knudsen, Carrillo, Swinnen, *et al.* 2016). This is in contrast to *S. cerevisiae* where glycerol uptake is solely mediated by the glycerol/H^+^ symporter Stl1 (Ferreira, van Voorst, Martins, Neves, Oliveira, *et al.* 2005). Therefore, we decided to investigate also the expression levels of genes putatively related to glycerol uptake.

**Figure 3:**
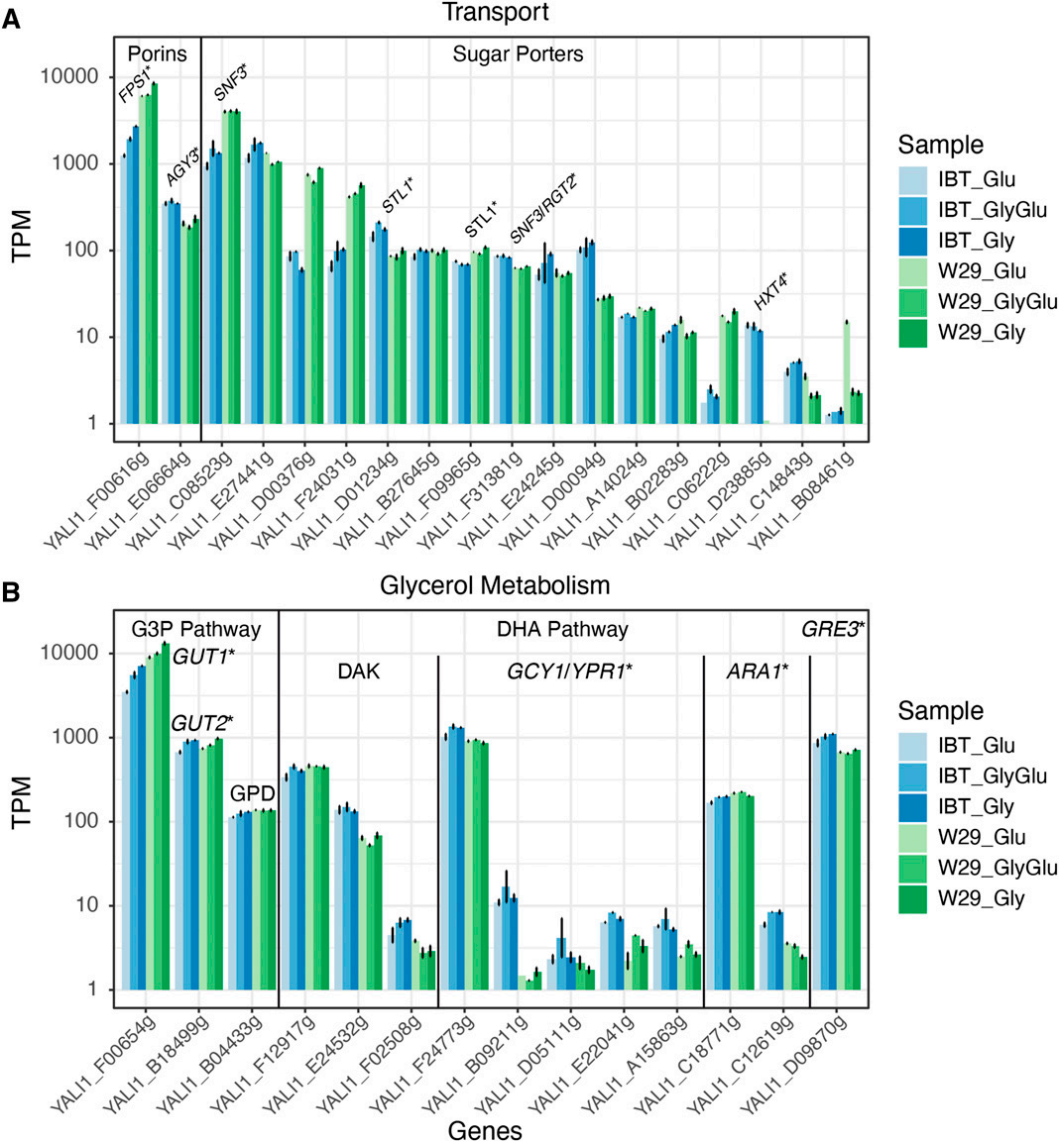
Results of the targeted transcriptome analysis. Expression levels are shown in log transcripts per million (logTPM) and names of *S. cerevisiae* orthologs are provided. (A) Expression of genes related to glycerol and sugar transport (see Table S1 for gene information). (B) Expression levels of genes related to glycerol metabolism (see Table S2 for gene information).

Raw count values were converted into transcripts per million (TPM) to allow for comparison of genes across samples (File S4). The expression levels of genes related to glycerol and sugar transport are provided in Figure 3A together with names of *S. cerevisiae* orthologs. Based on their level of expression, the *FPS1* ortholog *YlFPS1* (YALI1_F00616g), *YHT1* (YALI1_C08523g) and *YHT4* (YALI1_E27441g) are the dominatingly expressed transport related genes under the applied conditions. Interestingly, levels of *YlFPS1* and *YHT1* transcripts were significantly higher in W29 than in IBT. In both strains, expression of *YlFPS1* was strongly induced by glycerol, evidencing a transcriptionally-regulated role of this transporter in the assimilation of glycerol. Contrary, the expression of *YHT1* was not much affected by the various conditions in W29, whereas in IBT glycerol had a minor positive effect on its expression. *YHT4* was slightly higher expressed in IBT and also upregulated in the presence of glycerol, while in W29 this gene is majorly upregulated under glucose. Furthermore, two transporters YALI1_D00376g (class D) and YALI1_F24031g (class C) were nearly exclusively expressed in W29. For various putative transporter genes, expression levels were very low or absent in any of the tested conditions.

As stated above, we hypothesized that genes related to glucose transport or catabolism are subject to a glycerol induced repression in IBT but not in W29. In our study, however, we did not observe a repression on genes related to hexose transport. Nevertheless, the three transporters YALI1_C08523g (*YHT1*), YALI1_D00376g and YALI1_F24031g were significantly higher expressed in W29 throughout all conditions. This observation could potentially be related to the absence of glycerol attenuation in W29. Interestingly, Yht1 is closely related to the glucose sensors Snf3 and Rgts in *S. cerevisiae* (Lazar, Neuvéglise, Rossignol, Devillers, Morin, *et al.* 2017), while this would have to be further investigated to elucidate a potential relationship between these transporters and the observed physiological effects.

#### No evidence for glycerol repression observed in mRNA levels of glycolytic genes:

**Figure 4:**
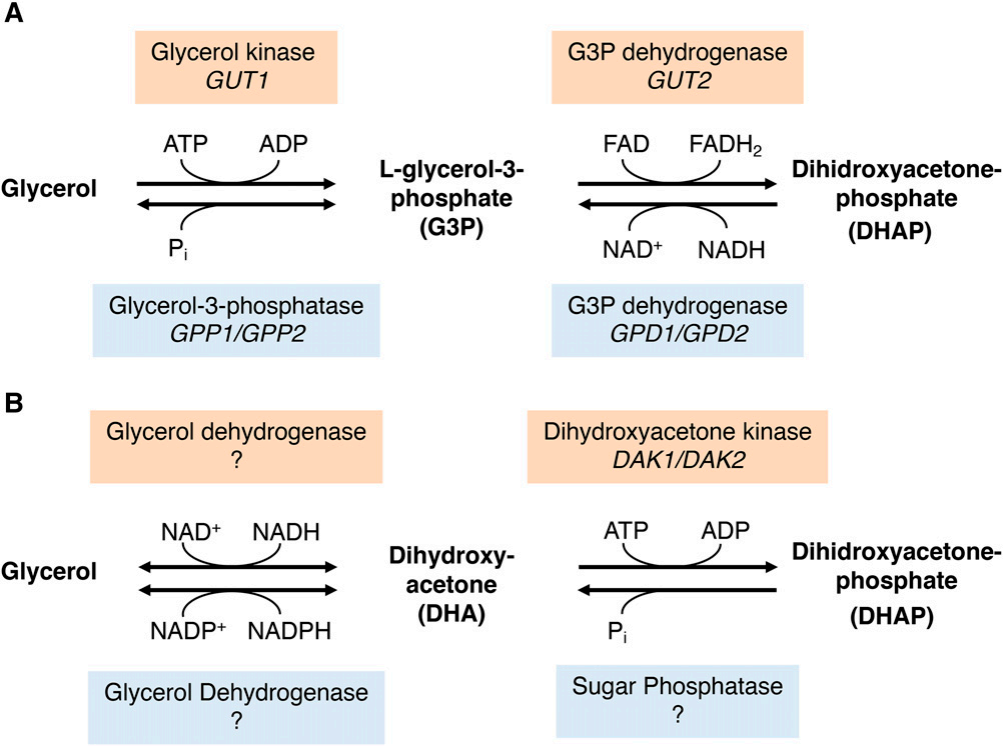
Glycerol catabolic (red) and anabolic (blue) pathways in yeasts. (A): G3P pathway. The catabolic G3P pathway starts with the phosphorylation of glycerol to glycerol-3-phosphate by the enzyme glycerol kinase (EC 2.7.1.30) followed by the oxidation to dihydroxyacetonephosphate (DHAP) by the mitochondrial membrane-bound enzyme glycerol-3-phosphate dehydrogenase (EC 1.1.5.3). As an intermediate of glycolysis/gluconeogenesis, DHAP enters the central carbon metabolism. In the anabolic G3P pathway, DHAP gets reduced to G3P by a cytosolic G3P dehydrogenase (EC 1.1.1.8/1.1.1.94) followed by the dephosphorylation of G3P to glycerol, catalyzed by the enzyme Glycerol-3-phosphatase (EC 3.1.3.21). (B): DHA pathway. The catabolic DHA pathway starts with the oxidation of glycerol to DHA by an NAD^+^-dependent glycerol dehydrogenase (EC 1.1.1.6) followed by a phosphorylation of DHA to DHAP by the dihydroxyacetone kinase (EC 2.7.1.29). In the anabolic DHA pathway DHAP is dephosphorylated to DHA by a so far uncharacterized sugar phosphatase (EC 3.1.3.23). DHA is subsequently reduced to glycerol by a NADP^+^-dependent glycerol dehydrogenase (GDH, EC 1.1.1.156). Confirmed *S. cerevisiae* genes are shown in italic, and *Y. lipolytica* orthologs are provided in Table S2.

Besides genes related to glucose transport, we hypothesized that genes involved in glucose catabolism could be other potential targets for a repression by glycerol. The catabolic routes of glycerol and glucose are connected via the intermediate DHAP (Figure 4). Therefore, we speculated that genes encoding enzymes of the upper glycolysis (before DHAP) could be repressed in IBT but not in W29. However, transcript levels of glycolytic genes revealed, that no significant downregulation under the investigated conditions occurred (data not shown).

#### Glycerol kinase YlGut1 shows the strongest expression among glycerol metabolic genes:

Next, we investigated the expression of genes related to glycerol metabolism in *Y. lipolytica*. As reviewed by Klein *et al.* (2017), two pathways exist for the metabolization of this compound in yeasts (Figure 4): the phosphorylative glycerol-3-phosphate pathway (G3P pathway) and the oxidative dihydroxyacetone pathway (DHA pathway). Both pathways can undergo two directions, depending on whether glycerol is used as a carbon source (catabolic route) or is synthesized to fulfill cellular functions (anabolic route). Glycerol metabolism has been investigated extensively in *S. cerevisiae* (Klein, Swinnen, Thevelein, and Nevoigt 2017), and genes from this species were used to identify corresponding orthologs in *Y. lipolytica* (Table S2). It is generally accepted that *Y. lipolytica* uses the glycerol-3-phosphate (G3P) pathway for glycerol consumption. As in *S. cerevisiae*, *Y. lipolytica* possesses single genes coding for glycerol kinase (*YlGUT1*, YALI1_F00654g) and mitochondrial G3P dehydrogenase (*YlGUT2*, YALI1_B18499g). Differences exist in the reverse enzymatic steps since only one cytosolic G3P dehydrogenase ortholog (*YlGPD1*) can be found in *Y. lipolytica* compared to two isogenes in *S. cerevisiae* (*GPD1*/*GPD2*). The cytosolic and mitochondrial G3P dehydrogenase isoforms are also participating to the so-called glycerol-3-phosphate shuttle (Dulermo and Nicaud 2011). Furthermore, no glycerol-3-phosphatase (GPP) ortholog could be identified in *Y. lipolytica* whereas *S. cerevisiae* again has two isogenes (*GPP1*/*GPP2*). An investigation by Mori *et al.* (2013) showed that Δ*YlGUT1* and Δ*YlGUT1*/Δ*YlGUT2* mutants of *Y. lipolytica* were strongly impaired in growth on glycerol, while a slight growth was still observable. The authors speculated that the faint growth could rely on an active catabolic DHA pathway. However, designating related genes by *in silico* predictions is challenging and the functions of related proteins often remain unknown. Even in *S. cerevisiae* the presence of an DHA pathway is still debated (Klein, Swinnen, Thevelein, and Nevoigt 2017): The strongest evidence has been the detection of significant dihydroxyacetone kinase (DAK) activity and the subsequent identification of corresponding genes (*DAK1*, *DAK2*), however, no *in vitro* activity of the glycerol dehydrogenase (first pathway step) has ever been measured in *S. cerevisiae* (Klein, Swinnen, Thevelein, and Nevoigt 2017), even while it was speculated that the genes *GCY1*, *YPR1*, *ARA1* or *GRE3* could catalyze this reaction (Izawa *et al.* 2004). *Y. lipolytica* possesses three orthologs of the dihydroxyacetone kinase, and interestingly, homology searches resulted in various homologs to *GCY1*, *YPR1*, *ARA1* or *GRE3*. Dulermo and Nicaud (2011) suggested these genes encode glycerol dehydrogenases participating in the DHA pathway.

In order to obtain a comprehensive picture of active genes related to glycerol metabolism, we compared their respective transcript levels (Figure 3B). During the applied conditions, glycerol kinase *YlGUT1* showed the strongest expression in both strains, while levels were nearly double in W29 compared to IBT. *YlGUT1* is furthermore strongly induced in the presence of glycerol in both strains. Expression of the glycerol-3-phosphate dehydrogenase, the second pathway step encoded by *YlGUT2* (YALI1_B18499g), was significantly lower and levels were similar in W29 and IBT. An inductive effect by glycerol was observable, however, much weaker compared to *YlGUT1*. Expression levels of *YlGPD1* (YALI1_B04433g) were even lower, with again similar values between both strains but no difference between the conditions. Expression of genes putatively related to the DHA pathway was detected. Two orthologs of DAK were expressed constitutively (YALI1_F12917g, YALI1_E24532g), however, the levels of the latter were very low. Three putative glycerol dehydrogenase orthologs (YALI1_F24773g, YALI1_D09870g, and YALI1_C18771g) were expressed, and levels of YALI1_F24773g were in the same magnitude as of *YlGUT2*. The expression of this gene was higher in IBT where it was also responsive to glycerol. The *GRE3* ortholog (YALI1_D09870g) exhibited a similar expression pattern and the *ARA1* ortholog (YALI1_C18771g) was only slightly expressed with similar expression levels throughout all conditions.

In summary, the transcriptome data confirmed prior studies suggesting glycerol catabolism is mediated by the G3P pathway in *Y. lipolytica* (Makri, Fakas, and Aggelis 2010; Dulermo and Nicaud 2011). Expression levels of *YlGut1* were significantly higher in W29 compared to IBT, which is potentially related to the higher respiratory rates in chemostat experiments. To the best of our knowledge, it has not been verified that *Y. lipolytica* contains an active DHA pathway. While several genes in its genome show similarities with glycerol dehydrogenases, these proteins require further functional characterization. As mentioned above, Dulermo and Nicaud (2011) classified the orthologs to S. cerevisiae GCY1, *YPR1*, *ARA1* or *GRE3* as glycerol dehydrogenases. Meanwhile, alternative functions other than the oxidation of glycerol have been annotated to several of these proteins. The putative glycerol dehydrogenases belong to the aldo-keto reductase (AKR) superfamily, whose enzymes have diverse functions in metabolism and their physiological roles are often unknown (Ellis 2002). It could be shown that YALI1_F24773g encodes an erythrose reductase, involved in erythritol biosynthesis (Janek, Dobrowolski, Biegalska, and Mirończuk 2017), and YALI1_D09870g is a xylose reductase (Ryu, Hipp, and Trinh 2015).

### Explorative transcriptome analysis: Using a hypothesis driven approach to detect global expression differences

**Figure 5:**
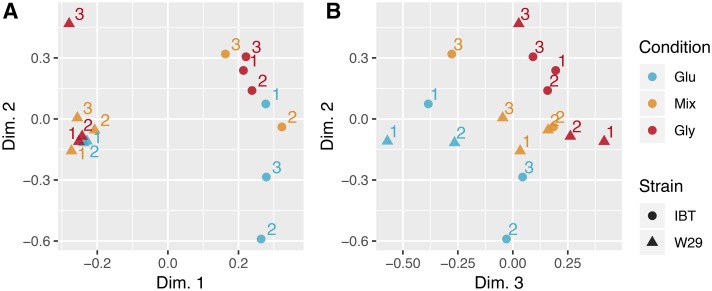
Singular Value Decomposition (SVD) plots showing separation by strains in dimension 1 *vs.* dimension 2 (A), and separation by growth condition in dimension 3 *vs.* dimension 2 (B). Numbers indicate the replicate within the strain and condition group. Dimensions 1-3 accounted for 47%, 14% and 10% of the total variance, respectively, in the RNA-Seq data set.

Glycerol repressive effects on genes related to glucose transport and catabolism could not be detected in the presented targeted analysis. To rather investigate global changes in transcriptional activity, we proceeded with a hypothesis driven explorative approach. The conducted RNA-Seq experiment represents a factorial design with the factors *strain* (W29, IBT) and *condition* (glucose, mix, glycerol). A Principal Component Analysis (PCA) revealed that most of the variance between the samples can be attributed to strain differences (Figure 5A), while the growth condition had a minor influence (Figure 5B). Furthermore, the response to the growth conditions occurred to be ordinal, largely following a linear trend (monotonic increase or decrease of glucose-mix-glycerol). The replicates IBT_Mix_1 and W29_Glucose_3 did not cluster together with the other samples and were excluded as outliers from the further analysis.

#### Almost 15% of genes vary expression level Between W29 and IBT:

To extract the strain effect (Figure 5A), a linear model was applied as detailed in Materials & Methods, identifying 1081 significantly differentially expressed genes (adj. p-value <0.05, |log2FC| <= 1). Resulting gene-level statistics of the linear model fit are provided in File S5. The differentially expressed genes were symmetrically distributed with approximately the same amount of up- and downregulated genes (553 and 528, respectively).

A gene-set analysis (GSA) was performed to facilitate the biological interpretation of affected differentially expressed genes (Table 3). Several processes were enriched, however, no coherent picture could be drawn from the results that indicate potential mechanisms behind the observed physiological differences: Genes of processes related to the translation machinery (including rRNA-, tRNA processing and ribosome biogenesis), oxidation-reduction processes and transport were enriched and mainly downregulated in W29 compared to IBT, while genes of the fatty acid metabolism, signal transduction and transcriptional regulation were mainly upregulated. Since the biological interpretation was complicated by the large number of differentially expressed genes affected by the strain differences we decided to investigate strain-specific responses to the carbon sources by cross-comparisons.

**Table 3:** Results of the gene-set analysis for the factor strain. Gene-sets have been manually curated to reduce redundancy and only gene sets with p-value < 0.05 are shown. The number of significant genes (p-value < 0.05) in a gene-set are provided together with the total amount of genes in the gene-set. Blue: gene sets containing mainly upregulated genes Up: gene sets containing mainly upregulated genes. Down: gene sets containing mainly downregulated genes

	Gene-set	Gene-set p-value	sig. genes	GO term
	acyl-CoA dehydrogenase activity	1.00E-04	11 / 11	GO:0003995
	1-phosphatidylinositol binding	1.45E-04	6 / 6	GO:0005545
	signal transduction	1.50E-04	44 / 72	GO:0007165
	fatty acid beta-oxidation	1.93E-04	8 / 8	GO:0006635
	DNA binding	2.00E-04	210 / 297	GO:0003677
**up**	protein heterodimerization activity	2.00E-04	20 / 28	GO:0046982
	small GTPase mediated signal transduction	2.50E-04	31 / 44	GO:0007264
	regulation of transcription, DNA-templated	5.50E-04	167 / 245	GO:0006355
	nucleosome assembly	9.00E-04	15 / 18	GO:0006334
	aminopeptidase activity	3.75E-03	10 / 12	GO:0004177
	oxidoreductase activity*^a^*	6.10E-03	9 / 12	GO:0016712
	RNA binding	1.00E-04	121 / 177	GO:0003723
	rRNA processing	1.00E-04	59 / 70	GO:0006364
	tRNA processing	1.00E-04	36 / 47	GO:0008033
	translation	1.50E-04	72 / 167	GO:0006412
**down**	oxidation-reduction process	2.88E-04	279 / 411	GO:0055114
	ATP-dependent helicase activity	3.50E-04	42 / 49	GO:0008026
	copper ion binding	1.00E-03	13 / 21	GO:0005507
	cell adhesion	4.47E-03	6 / 10	GO:0007155
	transmembrane transport	4.14E-02	211 / 308	GO:0055085

a gene-set name abbreviated.

#### Cross-comparisons revealed that strain-specific carbon source responses undergo in the opposite direction:

**Figure 6:**
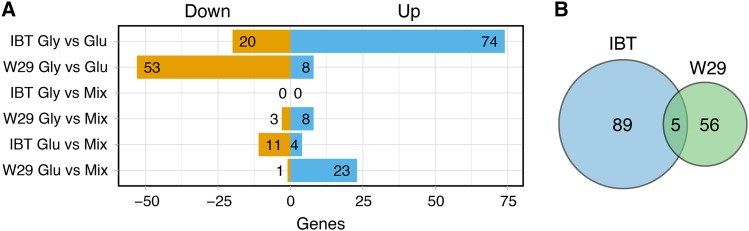
Cross comparisons between different samples of the same strain. (A) Significantly up and down regulated genes. (B) Intersection of differentially expressed genes in IBT and W29 by comparing the single carbon conditions glycerol *vs.* glucose.

Cross-comparisons between samples of the same strain were carried out in order to investigate strain-specific responses to the applied carbon conditions. A linear model was applied as detailed in the Materials & Methods section and gene-level statistics are provided in File S6. The number of regulated genes differed between the two strains, while unexpectedly the strain-specific carbon response regulated genes in opposite directions (Figure 6A). As anticipated, the largest effect on differential gene expression in both strains was observed by comparing the two single carbon conditions glycerol and glucose: In strain IBT, 94 genes were differentially expressed with the majority being upregulated, while in W29, 61 genes changed significantly under these conditions with the majority being downregulated. Among these genes, only five genes are shared between the strains (Figure 6B).

The comparison between glycerol and mixed condition resulted in the lowest number of differentially expressed genes, with only 11 genes significantly affected in W29 (from which six were also present in the glycerol *vs.* glucose contrast) and no significant genes in IBT. This signifies that the presence of glycerol in the mixed condition is dominant over the presence of glucose. Accordingly, the comparison between glucose and mixed conditions resulted in 15 significantly differentially expressed genes in IBT (from which 14 were also found in the glycerol *vs.* glucose comparison) and 24 genes in W29 (from which 23 were also in glycerol *vs.* glucose comparison).

Gene-set analyses for the glycerol *vs.* glucose comparisons have been carried out. The analysis indicated that the presence of glycerol upregulates various genes of processes related to nutrient scavenging in IBT, including the production of exoenzymes (proteases, lipases and glucosidases), transporters and oxidation-reduction processes (Table 4). Processes related to the gene expression machinery and DNA repair mechanisms were negatively affected. Contrary, the presence of glycerol seemed to downregulate lipases, proteases and genes related to oxidation-reduction processes in W29, revealing even similar processes have opposite responses in each strain (Table 5). Meanwhile, genes related to stress (starvation and filamentous growth), amino acid biosynthesis and transcriptional regulation were mainly upregulated. The direct comparisons within a strain revealed that in both strains not only cellular and metabolic processes were affected when grown on glycerol compared to glucose but also regulation was involved. Therefore, the next attempt was to investigate if there were significantly differences in the strains specific regulation.

**Table 4:** Results of the gene-set analysis for the direct comparison IBT glycerol *vs.* IBT glucose. Gene-sets have been manually curated to reduce redundancy and only gene sets with p-value < 0.05 are shown. The amount of significant genes (p-value < 0.05) in a gene-set are provided together with the total amount of genes in the gene-set. Up: gene sets containing mainly upregulated genes. Down: gene sets containing mainly downregulated genes

	Gene-set	Gene-set p-value	sig. genes	GO term
	amino acid transmembrane transport	1.00E-04	5 / 30	GO:0003333
	carbohydrate metabolic process	1.00E-04	8 / 69	GO:0005975
	oxidation-reduction process	1.00E-04	27 / 411	GO:0055114
	proteolysis	1.00E-04	10 / 176	GO:0006508
	pyridoxal phosphate binding	1.00E-04	10 / 51	GO:0030170
**up**	sequence-specific DNA binding RNA polymerase II transcription factor activity	1.00E-04	9 / 63	GO:0000981
	transmembrane transport	1.00E-04	27 / 308	GO:0055085
	transport	1.00E-04	20 / 332	GO:0006810
	glycerolipid metabolic process	1.44E-04	4 / 45	GO:0046486
	catalytic activity	1.50E-04	27 / 357	GO:0003824
	cell cycle	1.00E-04	5 / 59	GO:0007049
	cellular response to DNA damage stimulus	1.00E-04	4 / 37	GO:0006974
	DNA binding	1.00E-04	28 / 297	GO:0003677
	DNA repair	1.00E-04	6 / 92	GO:0006281
	helicase activity	1.00E-04	6 / 84	GO:0004386
**down**	methylation	1.00E-04	5 / 88	GO:0032259
	nucleic acid binding	1.00E-04	26 / 308	GO:0003676
	protein heterodimerization activity	1.00E-04	4 / 28	GO:0046982
	RNA binding	1.00E-04	8 / 177	GO:0003723
	RNA splicing	1.00E-04	6 / 38	GO:0008380
	damaged DNA binding	2.00E-04	4 / 14	GO:0003684

**Table 5:** Results of the gene-set analysis for the direct comparison W29 glycerol *vs.* W29 glucose. Gene-sets have been manually curated to reduce redundancy and only gene sets with p-value < 0.05 are shown. The amount of significant genes (p-value < 0.05) in a gene-set are provided together with the total amount of genes in the gene-set. Up: gene sets containing mainly upregulated genes. Down: gene sets containing mainly downregulated genes

	Gene-set	Gene-set p-value	sig. genes	GO term
	ATP catabolic process	1.00E-04	2 / 98	GO:0006200
	carbohydrate metabolic process	1.00E-04	4 / 69	GO:0005975
	cellular amino acid biosynthetic process	1.00E-04	3 / 34	GO:0008652
	filamentous growth of a population of unicellular organisms in response to starvation	1.00E-04	2 / 63	GO:0036170
**up**	lysine biosynthetic process	1.00E-04	2 / 8	GO:0009085
	phospholipid binding	1.00E-04	2 / 25	GO:0005543
	regulation of transcription from RNA polymerase II promoter	1.00E-04	8 / 104	GO:0006357
	regulation of transcription, DNA-templated	1.00E-04	11 / 245	GO:0006355
	zinc ion binding	1.00E-04	9 / 376	GO:0008270
	triglyceride lipase activity	1.50E-04	2 / 21	GO:0004806
	N-acetyltransferase activity	2.00E-04	3 / 29	GO:0008080
**down**	peptidase activity	6.00E-03	6 / 113	GO:0008233
	glucose transport	1.25E-02	1 / 6	GO:0015758
	oxidation-reduction process	1.34E-02	15 / 411	GO:0055114

#### Hypothesis-driven analyses highlights the involvement of regulatory proteins:

**Figure 7:**
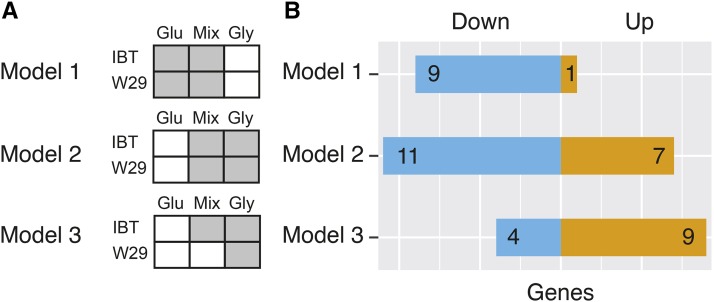
An approach to investigate differences in the carbon source specific regulation. (A): Illustration of the three models (hypotheses) used. (B): Amount of differentially expressed genes according the hypothesis tests related to model 1-3.

Cross-comparisons above indicated that IBT and W29 can have opposite response to nutrients. In an attempt to compare the strain-specific nutrient responses, we next analyzed all samples together. As illustrated in Figure 7A, three hypotheses were formulated and linear models applied accordingly (compare also the Materials & Methods section): We postulated that both strains possess genes which are responsive to the presence of glucose (model 1), while other genes are responsive to the presence of glycerol (model 2). As such, these two models focus on the most conserved response to nutrients, corresponding to Figure 5B. A third hypothesis was formulated to extract genes differently regulated in IBT and W29, where the expression in the mixed condition is in reverse between the strains (model 3).

By discarding the strain effect from the linear model (which results in high numbers of differentially expressed genes as shown above), the condition effect as defined in the three hypothesis appears to be rather small (Figure 7B). In total, ten genes are responsive to glucose in both strains (model 1), 18 genes are responsive to glycerol (model 2) and 13 genes respond differently in IBT and W29 (model 3). Gene-level statistics of the linear model fit are provided in File S7.

Model 3 represents the earlier defined hypothesis that genes exists which are differently regulated in IBT and W29. Interestingly, as shown in Table 6, several of the resulting genes are putatively related to transcriptional regulation (YALI1_A12929g, YALI1_A16891g) or signal transduction (YALI1_E01904g, YALI1_D22368g, YALI1_E14489g). Four of the genes are of unknown functions (YALI1_E24676g, YALI1_C13910g, YALI1_F38013g, and YALI1_C10173g) while two are mitochondrial genes (cob: cytochrome B, nad5: NADH-ubiquinone oxidoreductase chain 5).

**Table 6:** Significantly differentially expressed genes resulting from hypothesis 3 testing. Shown are the log2FC and P value from the linear model fit. Additionally, a blastp homology search has been conducted to receive protein functions from other yeast species. Up: gene sets containing mainly upregulated genes. Down: gene sets containing mainly downregulated genes

	YALI1 ID	YALI0 ID	log2FC	adj. P Value	E value	Identities	Description
	YALI1_A12929g	YALI0A12925g	3.66	0.002	2.00E-18	52/117 (44%)	similar to *S. cerevisiae* YGR044C *RME1* Zinc finger protein involved in control of meiosis
	YALI1_E01904g	YALI0E01364g	2.19	0.004	6.00E-82	148/393 (38%)	similar to *S. cerevisiae* YOR212W *STE4* G protein beta subunit, forms a dimer with Ste18p to activate the mating signaling pathway
	YALI1_D22368g	YALI0D18018g	1.78	0.004	2.00E-25	123/467 (26%)	similar to *S. cerevisiae SST2* (YLR452C) involved in desensitization to alpha-factor pheromone
	YALI1_E24676g	YALI0E20779g	1.57	0.034	NA	NA	no similarities
**up**	YALI1_E14489g	YALI0E11627g	1.55	0.004	2.00E-119	192/441 (44%)	similar to *S. stipitis* CBS 6054 guanine nucleotide-binding protein alpha subunit
	YALI1_A16891g	YALI0A16841g	1.51	0.006	3.00E-88	131/243 (54%)	similar to *S. cerevisiae* YOR113W *AZF1* Zinc-finger transcription factor
	YALI1_A11439g	YALI0A11473g	1.29	0.003	0.0	692/1266 (55%)	similar to *S. cerevisiae* YKL209C *STE6* Plasma membrane ATP-binding cassette (ABC) transporter required for the export of a-factor
	YALI1_C13910g	NA	1.04	0.005	NA	NA	no similarities
	YALI1_F38013g	YALI0F30437g	1.03	0.023	NA	NA	no similarities
	YALI1_E26094g	YALI0E22088g	−1.10	0.018	1.00E-18	50/107 (47%)	similar to *S. cerevisiae* YER011W *TIR1* Cell wall mannoprotein of the Srp1p/Tip1p family of serine-alanine-rich proteins
**down**	YALI1_C10173g	NA	−1.29	0.036	NA	NA	no similarities
	nad5	NA	−1.30	0.034	0.0	568/655 (87%)	*C. phangngaensis* NADH:ubiquinone oxidoreductase (mitochondrial gene)
	cob	NA	−2.81	0.013	0.0	264/385 (69%)	*K. marxianus* cytochrome b subunit of the bc complex (mitochondrial gene)

Expression profiles of differentially expressed genes as identified from model 3 are shown in Figure S2. YALI1_A12929g has similarity to *S. cerevisiae* Zinc finger protein Rme1 (YGR044C). This gene is not expressed in IBT, and in W29 expression under glycerol is significantly higher than under glucose and mixed conditions. In *S. cerevisiae* Rme1 is a nucleic-acid-binding protein that acts as a negative regulator of meiosis in haploid cells (a, α) but is repressed in diploid (a/α) cells (Covitz, Herskowitz, and Mitchell 1991). YALI1_E01904g shows a similar expression pattern, but is also slightly expressed in IBT. This gene has similarity with *S. cerevisiae* Ste4 (YOR212w), a GTP-binding protein subunit involved in pheromone-dependent signal transduction. As a part of a G protein heterodimer (Gβγ), Ste4 plays a critical role in the activation of several effector proteins (Henrik G. Dohlman and Thorner 2001). The homolog of another significant gene is also involved in the pheromone pathway: YALI1_D22368g, showing similarity to *S. cerevisiae* *SST2* (YLR452C), is lowly expressed in both strains, however, the expression is again enhanced in W29 grown on glycerol. In *S. cerevisiae* Sst2 is a member of the regulator of G protein signaling (RGS) family and negatively regulates pheromone response by stimulating GTP hydrolysis of the activated G protein α subunit (Gpa1) (Apanovitch, Slep, Sigler, and Dohlman 1998; H G Dohlman, Song, Ma, Courchesne, and Thorner 1996; Chan and Otte 1982). YALI1_E14489g exhibits an expression pattern similar to YALI1_E01904g and is homolog to a guanine nucleotide-binding protein alpha subunit in *S. stipitis* and other species. It is also homolog to *S. cerevisiae* Gpa2 (YER020W) which is part of a glucose sensing system together with the G-protein coupled receptor (GPCR) Gpr1 (Busti, Coccetti, Alberghina, and Vanoni 2010). YALI1_A11439g is expressed in both strains with slightly higher levels in W29. Again W29 grown on glycerol shows the strongest expression and the mixed condition behaves differently in IBT and W29. This gene is similar to S. cerevisiae STE6 (YKL209c), encoding an ATP-binding cassette (ABC) transporter protein, which mediates the export of the a-factor mating pheromone in *MATa* cells (Michaelis and Barrowman 2012). YALI1_A16891g is weakly similar to *S. cerevisiae* *AZF1* (YOR113w), encoding an asparagine-rich zinc finger protein. It is expressed in both strains with the highest expression under glycerol. IBT shows a linear increase of the expression from glucose to glycerol, while in W29 glucose and mixed conditions have similar expression levels. In *S. cerevisiae* Azf1 is a transcription factor responding to the specific carbon source present. Under glucose, genes involved in growth and carbon metabolism are activated, while the cell wall integrity is regulated in the presence of non-fermentable carbon sources (Slattery, Liko, and Heideman 2006). Lastly, YALI1_E26094g is also differently regulated in the two strains. It is upregulated in IBT on glycerol but downregulated in W29 under this condition. This gene is homolog to *TIR1* in *S. cerevisiae* which encodes a cell wall mannoprotein expressed under anaerobic conditions. It should be remembered that even though these genes have similarities with regulatory proteins in related species, it does not mean that their function is conserved. Further research is necessary to reveal the biological function of the identified proteins in *Y. lipolytica*.

### Conclusion

**Figure 8:**
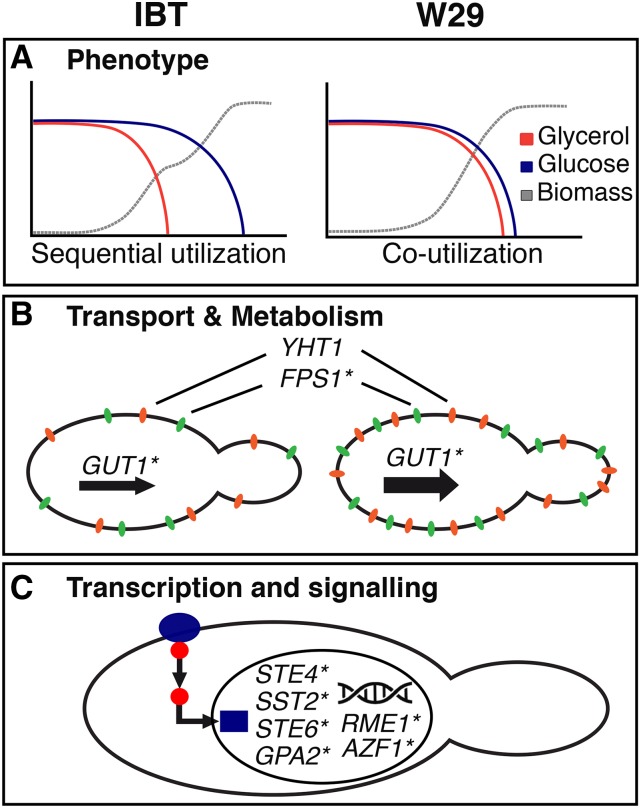
A summary of the main findings of this study. (A) Different phenotypes in the usage of glycerol and glucose have been detected between *Y. lipolytica* strain IBT (sequential utilization and diauxic behavior) and W29 (co-utilization and no diauxic behavior). (B) A hypothesized direct repression of glucose utilization genes has not been observed, however, several transporters (*YHT1* and FPS1*) and metabolic genes (GUT1*) were higher expressed in W29. (C) Finally, several genes encoding transcription factors and signal transduction proteins in *S. cerevisiae* were differentially expressed in the two strains. *S. cerevisiae* gene names are labeled with an asterisk symbol (*).

*Y. lipolytica* exhibits remarkable growth capabilities on glycerol, however, most of the current knowledge concerning glycerol uptake, catabolism and regulation is derived from *S. cerevisiae*, a yeast with natively limited abilities to utilize this substrate. *Y. lipolytica*’s glycerol utilization differs in several aspects from *S. cerevisiae*, while especially carbon source regulation is dissimilar. In contrast to regulatory mechanisms enabling the prioritized use of glucose, this non-conventional yeast prefers glycerol in co-substrate cultivations. This study has embarked on investigating not previously described carbon regulation in *Y. lipolytica*, by comparing the transcriptomes of strains differing in their substrate utilization phenotypes. Figure 8 summarizes the main findings of this study. Growth profiling demonstrated a strain-dependent physiology under glycerol-glucose mixed conditions, whose molecular background is so far unknown. Interestingly, transcriptome analysis revealed the majority of differentially expressed genes between the strains are regardless of the carbon source applied and no direct glycerol repression was observed for genes related to glucose uptake and catabolism in IBT. However, several genes were generally higher expressed in W29 including the transporters YALI1_F00616g (*YlFPS1)*, YALI1_C08523g (*YHT1)*, YALI1_D00376g, YALI1_F24031g and glycerol kinase YALI1_F00654g (*YlGUT1*). Different expression levels are potentially related to the observed substrate utilization phenotypes and has to be further investigated in future experiments. Even though no direct glycerol repression on genes related glucose degradation was detected in this study, it is feasible that such effects would be more prominent in different experimental designs. Previous results indicating the suppression of glucose in the presence of glycerol were obtained from batch cultivations, where high residual substrate concentrations can persistently induce relevant signaling pathways. In contrast, the expression profile data here was obtained from carbon limited chemostats where the substrate concentrations at all steady-state conditions were 0 g L^-1^. Nonetheless, cross comparisons did indicate transcriptional responses to the use of either carbon source during chemostat cultivations, while the genes affected in IBT and W29 were mostly different and their regulation was predominantly in opposite directions. This is signifying that regulation related to carbon source preference can also be observed in carbon limited chemostat cultivations. The analysis of differences in the carbon response revealed that several genes related to transcription factors and signal transduction are differently expressed between the strains. Orthologs of these genes are well known and involved in the mating pathway and carbon source regulation in *S. cerevisiae*. As such, this study lays the foundation for further investigations on carbon source regulation and glycerol repression-like effects in *Y. lipolytica*. Future work should include gene expression studies under batch conditions or additional chemostat setups, *e.g.*, with nitrogen limited conditions or substrate pulse experiments.
